# Near- and Far-Surround Suppression in Human Motion Discrimination

**DOI:** 10.3389/fnins.2018.00206

**Published:** 2018-03-29

**Authors:** Huan Wang, Zhengchun Wang, Yifeng Zhou, Tzvetomir Tzvetanov

**Affiliations:** ^1^Hefei National Laboratory for Physical Sciences at Microscale, School of Life Science, University of Science and Technology of China, Hefei, China; ^2^School of Medicine, Ningbo University, Ningbo, China; ^3^State Key Laboratory of Brain and Cognitive Science, Institute of Biophysics, Chinese Academy of Sciences, Beijing, China; ^4^Anhui Province Key Laboratory of Affective Computing and Advanced Intelligent Machine, and School of Computer and Information, Hefei University of Technology, Hefei, China

**Keywords:** near-surround, far-surround, spatial inhibition, motion repulsion, MT

## Abstract

The spatial context has strong effects on visual processing. Psychophysics and modeling studies have provided evidence that the surround context can systematically modulate the perception of center stimuli. For motion direction, these center-surround interactions are considered to come from spatio-directional interactions between direction of motion tuned neurons, which are attributed to the middle temporal (MT) area. Here, we investigated through psychophysics experiments on human subjects changes with spatial separation in center-surround inhibition and motion direction interactions. Center-surround motion repulsion effects were measured under near-and far-surround conditions. Using a simple physiological model of the repulsion effect we extracted theoretical population parameters of surround inhibition strength and tuning widths with spatial distance. All 11 subjects showed clear motion repulsion effects under the near-surround condition, while only 10 subjects showed clear motion repulsion effects under the far-surround condition. The model predicted human performance well. Surround inhibition under the near-surround condition was significantly stronger than that under the far-surround condition, and the tuning widths were smaller under the near-surround condition. These results demonstrate that spatial separation can both modulate the surround inhibition strength and surround to center tuning width.

## Introduction

Contextual interactions in visual processing are ubiquitous across a large panel of feature processing aspects and contribute to binding or segregating spatial elements in the visual field (Spillmann and Werner, 1996; Albright and Stoner, 2002; Kingdom et al., 2014; Tadin, 2015). The specific interaction patterns within a feature shed light on the underlying computational process. For example, the psychophysical results of modulating perception of orientation targets (e.g., perceived contrast changes, tilt illusion, or Vernier) are attributed to neuronal processing and interactions between orientation sensitive neurons with overlapping or non-overlapping receptive fields (Gilbert and Wiesel, 1990; Westheimer, 1990; Kapadia et al., 2000; Tzvetanov et al., 2007; Petrov and McKee, 2009; Tzvetanov, 2012), which are thought to reflect the early statistical structure of natural images (Yen and Finkel, 1998; Sigman et al., 2001; Simoncelli and Olshausen, 2001). In parallel, center-surround motion processing has demonstrated segregation and assimilation results (Murakami and Shimojo, 1995, 1996; Kim and Wilson, 1997; Tadin, 2015), which are associated with the hMT/V5 area of motion processing neurons and their interactions.

Psychophysics studies on center-surround motion processing have indicated that surround effects are selective for the motion direction, and the motion repulsion effect in this configuration is attributed to the suppressive interactions between motion sensitive neurons with non-overlapping receptive fields (Kim and Wilson, 1997; Tzvetanov and Womelsdorf, 2008; Tzvetanov, 2012). By applying center-surround configuration stimuli, the strongest suppressive effects appear when the surround motion has the same direction as the center target, and by contrast, surround stimuli facilitate neuronal responses to the center target if it moves in the opposite direction than that in the center (Allman et al., 1985a,b; Born and Tootell, 1992; Murakami and Shimojo, 1995, 1996). This effect is spatially selective, with the strongest effect found when the surround is adjacent to the center, and as the surround is moved further away, the suppressive effects decrease in amplitude, which explains the decrease in motion repulsion with center-surround separation (Kim and Wilson, 1997).

Recent evidence has demonstrated particular changes in orientation width modulation with the increasing spatial distance of the surround stimulation from the center. Shushruth et al. (2013) investigated the orientation tuning difference between near- and far-surround stimulation and showed that near-surround suppression has sharper orientation tuning than far-surround suppression for both macaque monkey V1 neurons and human perception. Based on the general similarity between motion and orientation contextual modulation (Tzvetanov, 2012), the above evidence provides an interesting possibility for the motion domain: although the amplitude of suppression decreases with spatial separation, distant motion surrounds should have broader motion direction tuning effects on the center. Thus, we investigated the influences of near- and far-surrounds using the motion repulsion effect as a probe of neuronal center-surround interactions. We extracted the individual human center-surround neuronal parameters of lateral inhibition and surround-to-center tuning width changes by fitting a computation model to the motion repulsion effects of each subject.

## Materials and methods

### Subjects

Eleven adult subjects (including two of the authors and 5 females aged 22–43 years) participated in the study. They had normal or corrected-to-normal vision. All subjects were naïve to the purpose of the experiment (excluding the two authors) and provided written consent for participating in the study. The experiment was approved by the Ethics Committee of the University of Science and Technology of China and conformed to the tenets of the Declaration of Helsinki.

### Apparatus and stimuli

Stimuli were generated with custom functions using the Psychtoolbox-3 (Brainard, 1997) and were displayed in the center of the screen, which was a (Sony MultiScan G520, Sony Corporation, Tokyo Japan) 21” CRT driven by an Nvidia Quadro K600 video card. The monitor parameters were as follows: 40.0 × 30.0 cm total display area, 1600 × 1200 pixel resolution, 85 Hz refresh rate, and 50.0 cd/m^2^ mean luminance. A circular window cut into a black cardboard centered on the screen, was used to limit the screen area to avoid local cues of vertical/horizontal positions (Tzvetanov, 2012). Subjects were seated in a dimly illuminated room and performed the task binocularly. The monitor was calibrated daily with a custom laboratory automated procedure.

Motion stimuli were moving black random-dot-patterns (RDPs) in a center-surround configuration with two surround conditions, which were called near- and far-surrounds. The center target motion had a 2° diameter, and the width of the surround annulus was 2° as well. For the far-surround condition, there was a blank annulus between the center target and surround context with a width of 2°; therefore, the total visual angle was 10°. However, the near-surround condition did not have a blank annulus, and the total visual angle was 6°. For both near- and far-surround conditions, the RDP had a density of 10 dots/square degree; each dot had a speed of 8°/s and a 0.1° diameter. The target motion stimulus in the center of the display had all dots moving in the same direction, which was maintained in a vertical upward direction with a staircase procedure. The surround direction of motion was defined with respect to the direction of the center target and had a variation range from −180° to 160° in steps of 20°; all of the surround dots had the same motion direction. In this experiment, a minus sign indicates counterclockwise from 0°, which was defined as a vertical upward motion.

### Procedure

Subjects performed the experiment after their head was stabilized with a chin-rest at a distance of 1.5 m from the screen. They were instructed to fixate on a fixation point (~0.067° size) displayed in the center of the screen throughout the entire measurement period, and the stimulus was centered at the fixation point. Each subject started the trial by pressing the spacebar on the keyboard, and 200 ms after the trial started, the whole center-surround stimulus was presented for 150 ms. Subjects were asked to report whether the center target direction was tilted clockwise/counterclockwise from the vertical upward direction in both near- and far-surround conditions. They answered by pressing one of two predefined keys on the keyboard. No feedback was provided to the subjects. Subjects experienced near- and far-surround conditions in random order. For the purpose of test-retest reliability, each subject performed the experiment twice.

A weighted up-down adaptive procedure (Kaernbach, 1991) was used for psychometric curve measurements. For each surround direction, two staircases were assigned with Up/Down steps of 3/1 and 1/3 steps of 1°, respectively, and each staircase contained 25 trials with a starting direction of +15°/−15°. Therefore, the measurements of the near-surround condition and far-surround condition included a total of 900 trials (18^*^2^*^25).

### Model application

The raw data of the near- and far-surround condition were fitted with a logistic function for every surround motion direction. The data consisted of computing the proportion of clockwise responses by using *p*_*i*_ = *y*_*i*_/*n*_*i*_, where *n*_*i*_ is the number of occurrences of the *x*_*i*_ target motion direction and *y*_*i*_ is the number of clockwise responses. The psychometric function was defined as:

(1) $$p\left(x\right)=l+\frac{1-2l}{1+exp\left(-\frac{log\left(21/4\right)}{\sigma }\left(x-\mu \right)\right)}$$

where *l* is the lapsing rate of the subjects, μ is the midpoint or perceived reference point, and σ is the threshold subjects needed to perceive a deviation from the reference point (that is, to arrive at 16 or 84% clockwise responses). This function was adjusted to the data using Bayesian fitting (Treutwein and Strasburger, 1999). The priors of the three parameters were:*l* – beta probability distribution with parameters Beta (1.2, 15), σ – gamma probability distribution with parameters Gamma (2.5, 2.5), and midpoint had uniform prior. After fitting, all of the midpoints of a given block of measures were adjusted to a mean of zero by subtracting their average.

The model of motion contextual interactions developed in previous research (Tzvetanov, 2012) was applied to infer the center-surround interaction parameters under near- and far-surround conditions (motion tuning widths and surround-to-center amplitude of inhibition). The model function that predicts the perceived motion direction is as follows:

(2) $$\begin{array}{l} Re^{j\theta_{pred}^{mo}}=\frac{1}{N}A_{max}[\sum_{i=1}^{n}e^{-\frac{{\left(\theta_{i}-\theta_{c}\right)}^{2}}{2\sigma_{c}^{2}}}e^{j\theta_{i}}\\ \;-A_{inh}\sum_{i=1}^{n}e^{-\frac{{\left(\theta_{i}-\theta_{s}\right)}^{2}}{2\sigma_{s}^{2}}}e^{-\frac{{\left(\theta_{i}-\theta_{c}\right)}^{2}}{2\sigma_{c}^{2}}}e^{j\theta_{i}}] \end{array}$$

where $Re^{j\theta_{pred}^{mo}}$ is the decoded motion obtained from the vector average across the population of neurons responding to the center stimulus; *N* is the sum of neuron responses (normalization factor); *n* is the number of neurons (*n* = 1440 for this study); θ_*c*_ and θ_*s*_ are the motion directions for the center and surround, respectively; *A*_*max*_ is the maximum firing rate of the center neuron tuned to its preferred input value θ_*i*_ under the circumstances of no surround being present; *A*_*inh*_ is the surround-to-center inhibition strength of the maximum response rate of the center neurons; σ_*c*_ is the motion tuning width of the center neuronal population; and σ_*s*_ is the motion direction tuning width of the surround-to-center inhibitory effect due to the presence of a surround with direction θ_*s*_.

Because of entanglement of the two tuning width parameters, and thus overfitting, their relation to each other was fixed in the near-surround condition. In the main text, the first results correspond to the condition of σ_*s*_ being equal to σ_*c*_ for the near-surround condition, and in the far-surround condition, σ_*c*_ was fixed at the value from the near-surround condition and σ_*s*_ was set as free. This allowed us to extract changes of the surround-to-center tuning width. The midpoint fitted from the psychometric function across different surround directions of motion was the parameter to be predicted by the model. The method of model fitting to the midpoint was as follows. For a given population characteristic (*A*_*inh*_, σ_*c*_), the predicted model directions of motion θ_*pred*_ were computed for all surround stimuli directions θ_*s*_ (θ_*c*_ fixed at 0 in the model). They were used in the sum-of -square equation:

(3) $$SSQ=\sum_{s=1}^{s=11}{\left(\mu^{s}-\mu_{pred}^{s}\right)}^{2}$$

where μ^*s*^ and $\mu_{pred}^{s}$ are the measured and predicted perceived vertical directions, respectively. The values for the amplitude of inhibition and tuning width that minimized the sum of squares (SSQ) were found with the help of the simplex algorithm. Given that 7 of the 11 subjects exhibited opposite surround attractive effects, which is not explainable by this model of inhibitory surround-to-center interactions, data for the surround angles larger than 100° were not included in the fitting (see also Tzvetanov and Womelsdorf, 2008; Tzvetanov, 2012). All of the statistical results were confirmed with different assumptions in the near surround condition: (1) σ_*s*_ = 1.5σ_*c*_, and (2) σ_*s*_ = σ_*c*_/1.5.

### Data analysis and statistics

First, the test-retest reliability between the first and second experimental run was analyzed for each subject. The raw repulsion effect was compared between the first and second run of each condition using Pearson's Product Moment correlation coefficient (similar results were found with linear fitting with error bars in both variables, Press et al., 1992). In the near-surround condition, the two measures were systematically strongly correlated (Pearson's *r*, mean 0.93, range [0.85:0.98], all *p* < 0.0001; see Supplementary Material-1) and demonstrated good reproducibility of the center-surround repulsion effect. For the far-surround condition, one of the 11 subjects did not show reliable test-retest reproducibility (*r* = 0.39, *p* = 0.11), while the remaining 10 subjects all had highly significant correlations (mean *r* = 0.85, range [0.66:0.96], all *p* < 0.004). The 11th subject, who did not show reliable reproducibility, was also among the subjects with the smallest repulsion effect for all conditions, and one far-surround data set did not exhibit a repulsion effect (see *F*-test below). Thus, the far-surround condition included only 10 subjects' data set.

Then, for each subject and each run, the model fit was compared to the global mean of the data by using an *F*-test that allowed an estimation of whether the model predicted the data better than the mean, i.e., whether there was any repulsion effect. The *F*-test was implemented as:

(4) $$F=\frac{\left(SS_{mean}-SS_{model}\right)/\left(df_{mean}-df_{model}\right)}{\left(SS_{model}/df_{model}\right)}$$

where *SS* is the residual sum of squares and *df* is the degrees of freedom.

Finally, a paired *t*-test was performed to compare the change or not of the surround-to-center inhibition strength *A*_*inh*_ and surround-to-center tuning width σ_*s*_ under near- and far-surround conditions. Since each subject completed the task two times, the mean values of the first and second run for each variable were calculated.

## Results

**Figure 1:**
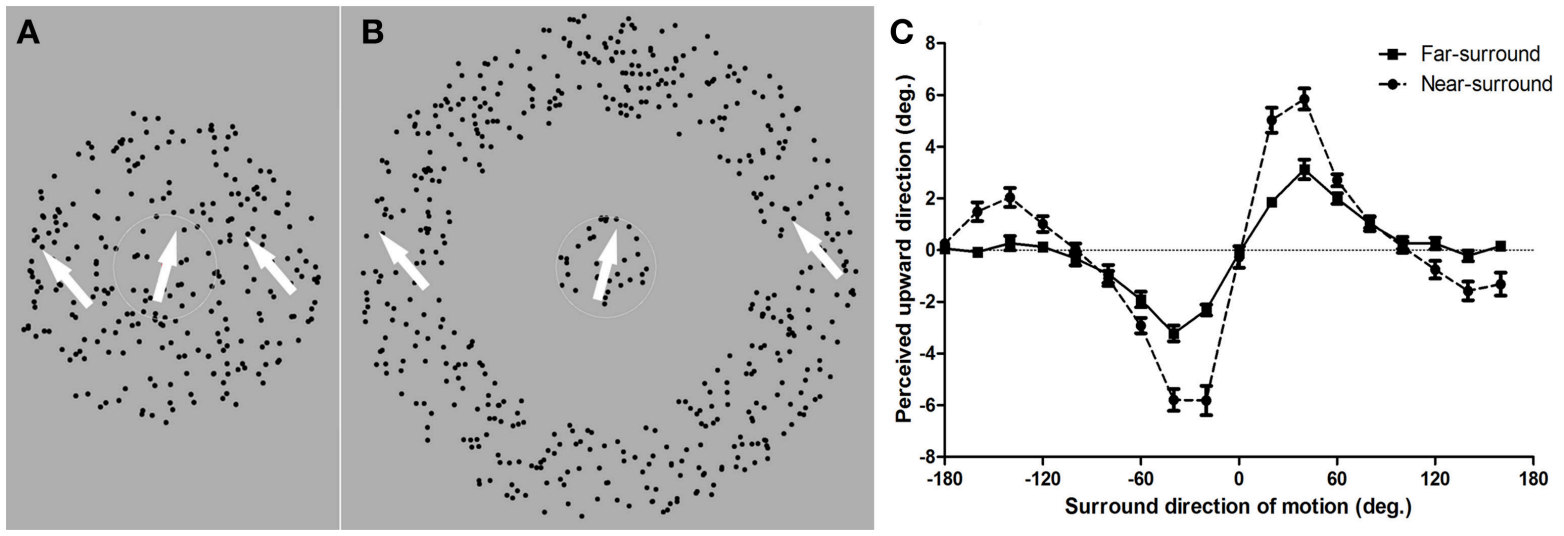
Diagram of random dot pattern stimuli in near- and far-surround motion discrimination tasks and mean repulsion effects. **(A)** Near-surround condition. Dots in the center area moved in different directions compared to those in the surround area. Subjects were required to report whether the motion direction of the center dots was clockwise or counter clockwise from his/her internal vertical standard by pressing two predefined keys. **(B)** Far-surround condition. The size of the center area was equal to that in the near-surround condition. A blank annulus width that was identical to the surround area in the near-surround condition was displayed. Subjects judged the direction of the center RDP. The white arrow indicates the motion direction of the corresponding part of the RDP. **(C)** Mean repulsion effect for the near- and far-surround conditions. The target direction of motion perceived as upward vertical motion is plotted as a function of the surround motion direction. Zero degrees is upward motion and positive values are clockwise tilts from zero. Error bars depict the SEM (*n* = 11 for near-surround; *n* = 10 for far-surround).

We measured human subjects' motion repulsion effects under near- and far-surround center-surround configurations of stimuli (Figures 1A,B). We further determined the changes in the surround inhibition strength and tuning widths with the surround distance by fitting a computational model to the behavioral data.

**Figure 2:**
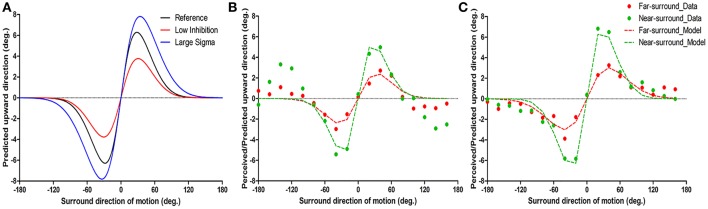
Example of model predictions **(A)** and of typical motion repulsion effects in two subjects and the model results **(B,C)**. The ordinate is the physical direction of the center target motion predicted/perceived as upward vertical motion, and the abscissa is the surround direction of motion. **(A)** The two parameters have different influences on the shape of motion repulsion tuning curve. The black line is the reference tuning curve with *A*_*inh*_ = 0.6 and σ_*s*_ = 25, the red curve has lower *A*_*inh*_ (0.4) and the blue curve has larger σ_*s*_ (30) than the black repulsion curve. **(B,C)** The results of psychophysics and model fitting are shown together and are indicated as dots and dashed lines separately. Individual data are the mean of the first and second experimental runs. **(B)** Psychophysical results show repulsion near ± 20–60° surrounds, while around ± 120–160° surrounds, an attraction effect was systematically present; note that while the model structure cannot predict attraction effects, it consistently fitted the direct repulsion effect. **(C)** Instance of a simple repulsion effect without opponent motion effects.

A motion repulsion effect, as reported in previous research (Kim and Wilson, 1997; Tzvetanov and Womelsdorf, 2008), was found for all 11 subjects under the near-surround condition (Figure 1C, dots). The motion direction that was, on average, perceived as moving upward vertically was strongly modulated by surround directions of ±20, ±40, and ±60°, with a peak misperception around ±20–40° surround directions. Additionally, an attraction effect occurred for surround directions of ±120–160° and were clearly present in individual data for 7 of the 11 subjects (examples are shown in Figures 2B,C). This opponent motion effect is known in the literature (Tzvetanov and Womelsdorf, 2008) and was not investigated further here since we were interested in the repulsion effect for center-surround suppressive effects close to the motion direction of the center.

The far-surround condition showed clear motion repulsion effects for 10 subjects (see Supplementary Material-1). The mean data of these subjects had a similar shape as those of near-surround condition, but with a much smaller amplitude of repulsion (Figure 1C, squares). Motion surround directions of ±20, ±40, and ±60° from the central motion showed the strongest modulations.

To understand the putative neuronal changes in the center-surround effects with surround distance, we used a computationally simplified theoretical model of center-surround interactions in motion processing (Tzvetanov and Womelsdorf, 2008; Tzvetanov, 2012) that allowed us to predict the direct repulsion effect for surround motions near the target direction (within ±100°). The two major parameters of the model influence the shape of motion repulsion curve (peak position and amplitude) in different ways. Figure 2A shows how motion repulsion effects change with variation of a single parameter. Surround-to-center inhibition strength *A*_*inh*_ mainly affects the peak amplitude of repulsion curve (Figure 2A, compare black and red curves; decreasing *A*_*inh*_ mainly reduced the amplitude of repulsion). In contrast, variation in center tuning width σ_*c*_ affects both the peak position and amplitude of repulsion (Figure 2A, compare black and blue curves; increasing σ_*s*_ shifted the peak position and increased the amplitude; here assuming σ_*c*_ = σ_*s*_).

**Figure 3:**
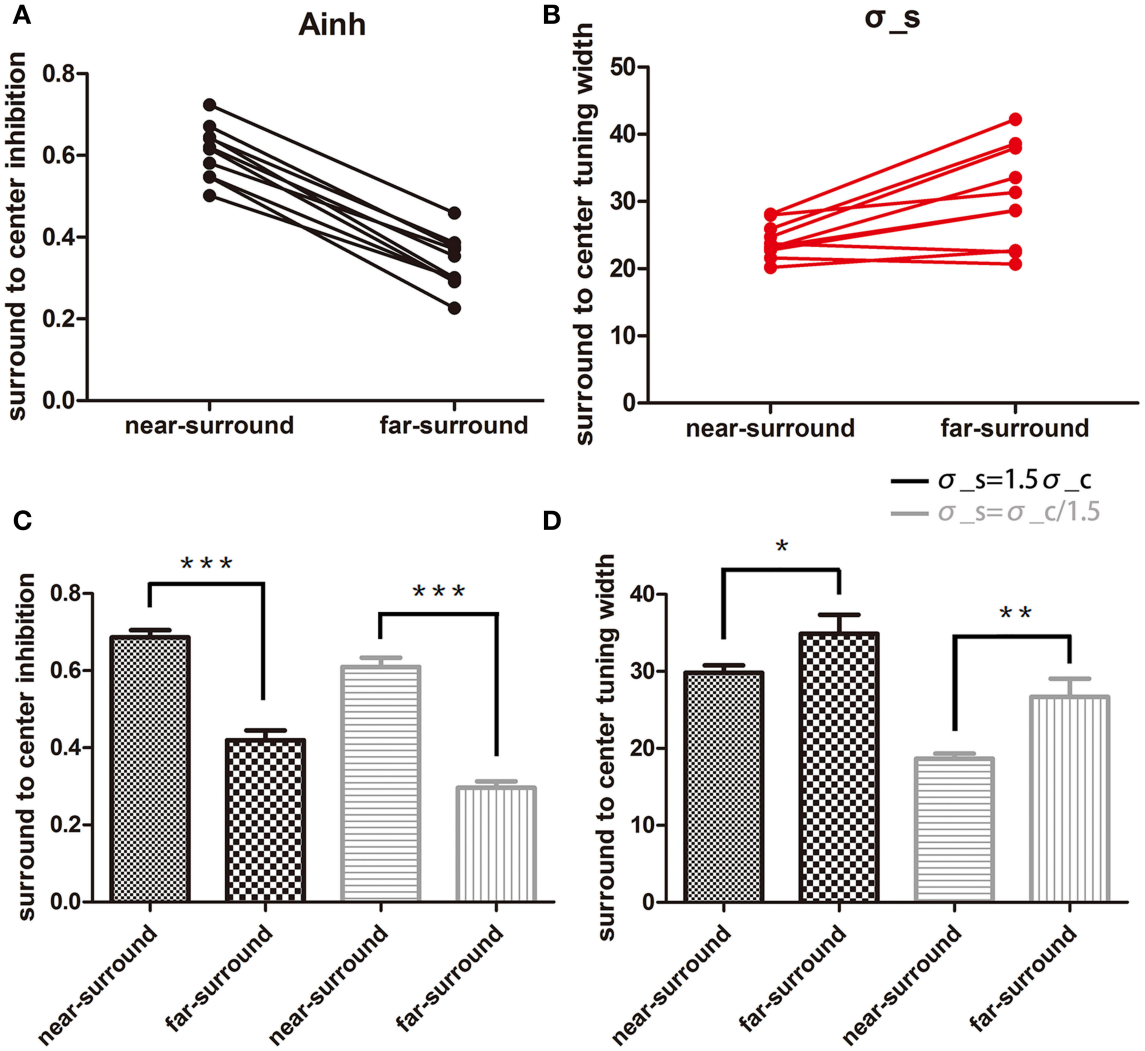
Comparison of changes in the two model parameters. **(A,B)** Plots of surround-to-center inhibition strength (*A*_*inh*_) and surround-to-center tuning width (σ_*s*_) for two conditions under assumption of σ_*s*_ = σ_*c*_. Each dot represents one subject, and only 10 subjects' data were used for a paired *t*-test. **(C,D)** Statistical results for the two assumptions of σ_*s*_ = 1.5σ_*c*_ and σ_*s*_ = σ_*c*_/1.5. *A*_*inh*_ is shown in **(C)**, and σ_*s*_ is shown in **(D)**. Black and gray bars are for assumptions of σ_*s*_ = 1.5σ_*c*_ and σ_*s*_ = σ_*c*_/1.5, respectively. Error bars are the SEM. ^*^p < 0.05; ^**^p < 0.01; ^***^p < 0.0001.

The near-surround condition permitted extraction of two major parameters: *A*_*inh*_ and σ_*c*_ (assuming equality of the center tuning width and surround-to-center tuning widths, see Methods and further below). First, we found that the model was able to predict all of the direct repulsion effects in the near-surround condition of the 11 subjects well (for details, see Supplementary Material-2). Figures 2B,C depict two subjects' individual repulsion curves and the model fits. The example provided in Figure 2B presents the subject's data with the direct and strongest opposite direction effects for the near-surround condition (green dots). The subject's data depicted in Figure 2C show that there was a simple direct repulsion effect without opposite surround effects. In general, the model successfully predicted subjects' motion misperceptions for surround directions close to the center motion. Across subjects, the mean surround amplitude of inhibition was 0.59 ± 0.03 (*n* = 11) and the tuning width was 24.6 ± 0.87° (*n* = 11) (Figures 3A,B).

Under the far-surround condition, the model allowed the surround amplitude of inhibition and surround-to-center tuning width to be extracted, or their relative changes, by using the individual fit results of the near-surround condition. Under this far-surround condition, we found that the model predicted the direct repulsion effects of 10 of the 11 subjects well (for details, see Supplementary Material-2). Figures 2B,C presents the two example subjects' far-surround condition data and model fits, showing the relatively good model prediction of the motion repulsion curve in the far-surround condition. Under this condition, across subjects, the mean amplitude of inhibition was 0.34 ± 0.02 (*n* = 10) and mean surround-to-center tuning width was 30.7 ± 2.6° (*n* = 10).

Comparing the results of the near- to far-surround conditions resulted in significant decreases in the amplitude of inhibition [*t*_(9)_ = 18.1, *p* < 0.0001, Figure 3A) and a significant increase in the surround-to-center tuning widths [*t*_(9)_ = 3.59, *p* < 0.05, Figure 3B].

The above results are based on the near-surround condition surround-to-center tuning modulation being equal to center tuning width. But the exact relation between these two values might be different, especially since physiological results showed slightly larger surround tuning widths than the center (Born, 2000). Therefore, we also tested how different assumptions affect the statistical outcome. The above results were confirmed when the model for the near-surround condition had different assumptions of center-surround tuning widths: (1) broader surround-to-center tuning width σ_*s*_ = 1.5σ_*c*_, (2) narrower surround-to-center tuning width σ_*s*_ = σ_*c*_/1.5. Both assumptions showed consistent results with the case σ_*s*_ = σ_*c*_. Figures 3C,D show the results of *A*_*inh*_ and σ_*s*_ under each assumptions and conditions. For σ_*s*_ = 1.5σ_*c*_, *A*_*inh*_ in the near-surround was significantly larger than that in the far-surround [0.69 ± 0.02, 0.42 ± 0.03, *t*_(9)_ = 13.9, *p* < 0.0001] and σ_*s*_ in the near-surround was significantly smaller than that in the far-surround [29.8 ± 0.99, 34.9 ± 2.48, *t*_(9)_ = 2.81, *p* = 0.0204]. For σ_*s*_ = σ_*c*_/1.5, the near-surround condition had a larger *A*_*inh*_ [0.61 ± 0.02, 0.30 ± 0.02, *t*_(9)_ = 21.2, *p* < 0.0001] and smaller σ_*s*_ [18.7 ± 0.65, 26.7 ± 2.37, *t*_(9)_ = 4.05, *p* = 0.0029] than the far-surround condition.

## Discussion

In this study, we found that near- and far-surround motion discrimination tasks led to different motion repulsion results. The motion repulsion effects caused by the near-surround condition were stronger than those in the far-surround condition, as reported previously (Kim and Wilson, 1997). A simple physiological model of the repulsion effect provided a good account of the results and further demonstrated that the increase in distance between the surround and center not only decreased the inhibitory effect of the surround onto the center but, importantly, also increased the surround to center tuning width of inhibition.

Various behavioral measures support the concept of suppressive surround-to-center motion interactions. In one type of research, when using simple grating stimuli, it was found that human perceptual performance was decreased by high contrast and large sized motion stimuli (Tadin et al., 2003; Paffen et al., 2005; Glasser and Tadin, 2011; Turkozer et al., 2016). Similarly, it was reported that assimilation or segregation of spatially separated moving random-dot-patterns (Nawrot and Sekuler, 1990; Murakami and Shimojo, 1996) were dependent on the sizes of the inducing and target areas. When the data were scaled for eccentricity effects, the results followed a general pattern of modulation that recalled classic center-surround receptive field responses with increasing stimulus size (Murakami and Shimojo, 1995, 1996), with maximum suppressive/segregation effects observed within some restricted range of the center sizes. Last, using the perceived motion direction of a central pattern as a probe, it was found that surround motions created repulsive effects on the perceived center direction (Kim and Wilson, 1997; Tzvetanov and Womelsdorf, 2008), an effect that faded with increasing spatial separation.

In these behavioral and modeling studies, the way that the surround modulates the center as a function of their separation was assumed to be a simple decaying amplitude effect. Our results further showed that, in addition, the direction width of the surround modulation increased with spatial distance, similar to that reported in the orientation domain (Shushruth et al., 2013). Because the visual system is known to learn the statistical structure of the visual input, the tuning width effects in both feature domains should correlate to equivalent statistical changes of the visual inputs between spatially distant patterns (orientation or motion direction), an effect that, to our knowledge, has not yet been reported.

Researchers have reported that contextual modulation in the motion domain can be either antagonistic or integrative, which strongly depends upon the visual stimulus design and parameters (Nawrot and Sekuler, 1990; Braddick, 1993; Murakami and Shimojo, 1993, 1995, 1996; Huang et al., 2007, 2008). For example, Huang et al. (2007, 2008) found that when using the same contextual contour, if the target stimuli were closely matching contours surround modulation was mostly integrative, while on the contrary if target stimuli were random-dot-patterns the effect was predominately antagonistic modulation. Furthermore, their psychophysics data were consistent with the neurophysiological results (Huang et al., 2008), nicely demonstrating surround integration and segregation dependence on contextual similarity between “center” and contextual features. In our study, we found surround attractive effects at surround directions of ±120–160°For 7 of 11 subjects under near-surround condition. However, the current model can not account for these attractive effects since it is based on simple inhibitory interactions between similarly tuned motion direction neuronal populations. The model could be extended further, as in Gilbert and Wiesel (1990), in order to predict these attractive effects but this would still leave unexplained the nature of presence or not of the effect across different subjects (see also Tzvetanov and Womelsdorf, 2008). Therefore, we mainly concentrated in this study on the direct repulsive effects of surrounds with directions of motion close to the central target, which showed a consistent pattern of modulation across observers and with spatial distance.

Interestingly, the behavioral results in the orientation domain were supported by equivalently carried neurophysiological measures (Shushruth et al., 2013). To explain the different orientation tunings of near- and far-surround suppression, the authors proposed that their observations were shaped by different cortical circuits of the primary visual cortex (V1) or feedback connections (Angelucci et al., 2002; Angelucci and Bressloff, 2006). The feedforward connections from the LGN to V1 (Ozeki et al., 2004) and intra-V1 horizontal connections contribute to the modulation between the cRF center and near-surround, while far-surround suppression arises from feedback connections from the extrastriate cortex to V1 (Girard et al., 2001; Angelucci and Bressloff, 2006; Shushruth et al., 2009; Angelucci and Shushruth, 2013; Nurminen and Angelucci, 2014). Therefore, broader orientation tuning of far-surround suppression indicated that feedback connections are more broadly orientation tuned than local intracortical connections.

Based on the similarity of center-surround effects in the motion direction and orientation features (Tzvetanov, 2012), we hypothesize that the direction tuning changes of surround suppression are due to different cortical circuits of the MT visual area and feedback interactions from the motion sensitive area MST onto the MT area. Because center-surround neurons were found in all layers of the MT but were less pronounced in layer IV (Raiguel et al., 1995; Born, 2000), it was suggested that local surround inhibition was mediated by intra-MT connections, which should be sharply tuned. By contrast, MST-to-MT feedback projections should be less direction specific, in a similar fashion as V1 feedback projections, for the simple reason that MST neurons encode more complex motion statistics as optic flow patterns (Krause and Pack, 2014).

## Author contributions

Conception and design of the experiments: TT, HW, and ZW. Acquisition of simulation and behavioral data: HW and TT. Analysis of data: HW and TT. Critical revisions of the paper and approval of the final version: HW, ZW, YZ, and TT.

### Conflict of interest statement

The authors declare that the research was conducted in the absence of any commercial or financial relationships that could be construed as a potential conflict of interest.
