# Supplementary material for: Near- and Far-Surround Suppression in Human Motion Discrimination
Source: Front Neurosci. 2018 Mar 29;12:206. doi: 10.3389/fnins.2018.00206 (PMC5884933; doi:10.3389/fnins.2018.00206)
Supplement: Supplementary file 1 [file DataSheet1.pdf]

Supplementary Material

Near- and far-surround suppression in human motion discrimination

Huan Wang, Zhengchun Wang, Yifeng Zhou , Tzvetomir Tzvetanov *

*** Correspondence:**
Tzvetomir Tzvetanov
E-mail: tzvetan@hfut.edu.cn.

1. Test-retest analysis

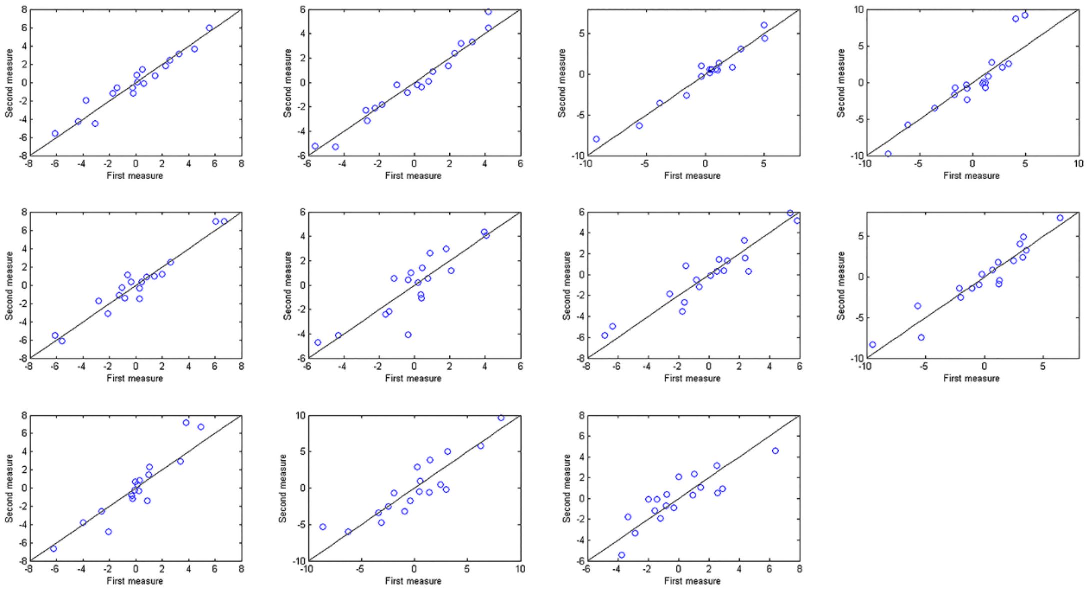

Supplementary Figure 1. Pearson correlation plots for every subject under the near-surround condition. Each panel stands for the perceived vertical motion of one subject. The ordinate is the second experimental measure, and the abscissa is the first measure. The solid diagonal lines indicate the line of equality (first measure = second measure). Each blue circle indicates a data point from one surround motion direction. All subjects showed good correlation.

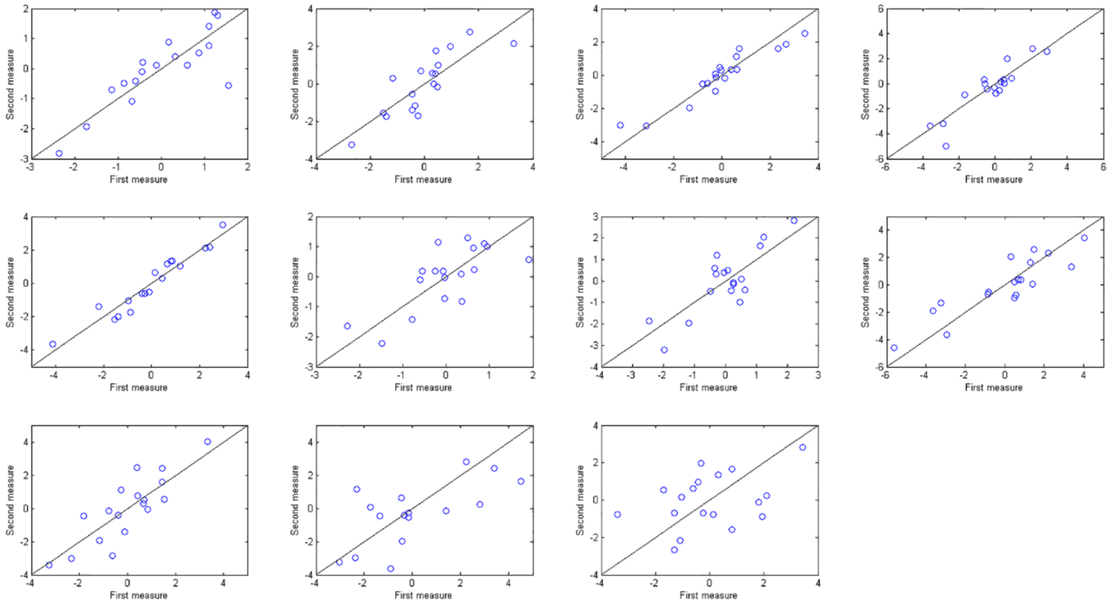

Supplementary Figure 2. Correlation between the 1st and 2nd measure of the far-surround condition. All plotting conventions are as in Figure S1. Only the 11th subject had a non-significant correlation.

Supplementary Tables 1. Pearson correlation coefficients

Subjects	Near-surround		Far-surround	
	r	p	r	p
1	0.965	9.55E-11	0.836	1.54E-05
2	0.981	7.93E-13	0.838	1.43E-05
3	0.977	3.97E-12	0.949	2.05E-09
4	0.925	4.08E-08	0.939	8.19E-09
5	0.967	5.77E-11	0.963	1.50E-10
6	0.870	2.73E-06	0.731	0.001
7	0.938	9.46E-09	0.827	2.36E-05
8	0.953	9.80E-10	0.884	1.11E-06
9	0.938	9.49E-09	0.846	9.70E-06
10	0.896	4.85E-07	0.657	0.003
11	0.846	9.56E-06	0.390	0.109

2. Results of the F-test of residual variance between the data means and model predictions.

Supplementary Tables 2. Results of the F-test

Subjects	First run				Second run			
	Far-surround		Near-surround		Far-surround		Near-surround	
	F(2,15)	p	F(2,15)	p	F(2,15)	p	F(2,15)	p
1	10.3	0.001	13.5	4.41E-04	46.9	3.53E-07	17.9	1.06E-04
2	41.0	8.31E-07	15.7	2.13E-04	12.1	7.33E-04	18.9	7.95E-05
3	86.7	5.72E-09	39.2	1.11E-06	42.8	6.29E-07	51.3	1.97E-07
4	80.3	9.67E-09	41.2	8.05E-07	83.3	7.52E-09	86.4	5.87E-09
5	75.2	1.52E-08	107	1.32E-09	50.4	2.21E-07	137	2.28E-10
6	18.0	1.04E-04	58.5	8.23E-08	7.83	0.005	13.7	4.18E-04
7	49.6	2.45E-07	39.4	1.07E-06	33.4	2.96E-06	60.8	6.39E-08
8	65.4	3.90E-08	29.0	9.52E-09	23.2	2.56E-05	42.9	6.25E-07
9	42.8	6.28E-07	80.5	9.52E-09	40.0	9.67E-07	87.9	5.21E-09
10	14.7	2.91E-04	33.7	2.85E-06	32.5	3.54E-06	24.2	2.01E-05
11	2.32	0.132	21.5	3.98E-05	11.7	8.51E-04	8.91	0.003

Only one subject (No. 11) had no significant model prediction under the first run far-surround condition.
